# Cervical Diffuse Idiopathic Skeletal Hyperostosis (DISH) as an Underrecognized Cause of Dysphagia: A Case Series and Review of the Literature

**DOI:** 10.7759/cureus.97249

**Published:** 2025-11-19

**Authors:** Emmanouela Dionysia Laskaratou, Ioannis Sperelakis, Nikolaos Trygonis, Rozalia Dimitriou, Georgios Kontakis

**Affiliations:** 1 Medical School, University of Crete, Heraklion, GRC; 2 Orthopaedics and Traumatology, University Hospital of Heraklion, Heraklion, GRC; 3 Hospital Medicine, Klinik für Orthopädie, Hand- und Unfallchirurgie, Stadtspital Zürich, Zurich, CHE; 4 Orthopaedics, University of Crete, Heraklion, GRC

**Keywords:** case series, cervical osteophytes, diffuse idiopathic skeletal hyperostosis, dysphagia, literature review

## Abstract

Diffuse idiopathic skeletal hyperostosis (DISH) represents an uncommon but clinically significant cause of secondary dysphagia. Ιn this paper, we report three elderly male patients presenting with progressive dysphagia secondary to cervical DISH. Diagnostic imaging confirmed prominent anterior cervical osteophytes at C3-C5. All patients underwent successful anterior osteophyte excision with complete symptom resolution. A review of 39 published cases from the last decade highlights diagnostic pitfalls, optimal imaging, and surgical considerations. Cervical DISH should be suspected in elderly patients with unexplained dysphagia, as timely recognition and surgical management can provide excellent outcomes.

## Introduction

Diffuse idiopathic skeletal hyperostosis (DISH), also known as Forestier’s disease, is a systemic, non-inflammatory condition characterized by flowing ossification of the anterior longitudinal ligament and formation of at least three contiguous bony bridges along the anterolateral aspect of the spine, most commonly in the thoracic and cervical regions [1]. Its reported prevalence varies widely across populations, from 2.9% in Korea to up to 42% in the United States, and it predominantly affects elderly males [2-5]. Although DISH is often asymptomatic or presents with musculoskeletal complaints, cervical involvement may rarely result in compressive symptoms.

DISH should be differentiated from ankylosing spondylitis, which may present with spinal stiffness, but is typically seen in younger patients and is characterized by sacroiliac joint involvement, thin marginal syndesmophytes, and inflammatory features. In contrast, DISH shows flowing anterolateral ossification with preserved disc spaces and apophyseal joints [1].

When large anterior cervical osteophytes develop, they may impinge on the pharyngo-esophageal tract and cause secondary dysphagia. Cervical osteophytes are estimated to account for approximately 1.7% of all dysphagia cases, making this an uncommon but clinically important etiology, particularly in older adults [6]. Most published cases over the past decade originate from Asian countries (Korea, Thailand, Iran, India, Japan), suggesting either regional reporting patterns or under-recognition in Western populations [7-11]. Besides dysphagia, aspiration pneumonia represents the most frequent associated complication, while stridor, cervical pain, and, much more rarely, cervical myelopathy have also been described [8,11,12].

Diagnosis is often delayed because symptoms are non-specific and patients are initially evaluated by ENT, gastroenterology, or general surgery services. In many instances, DISH-related dysphagia is established only after more common causes have been excluded. Imaging of the cervical spine (plain radiographs, CT, MRI) typically reveals prominent osteophytosis at C3-C5, where the esophagus lies in close proximity to the anterior cervical spine, providing a clear anatomical explanation for swallowing difficulty. Conservative management, including dietary modification and anti-inflammatory or muscle-relaxant medication, is generally considered first-line, whereas surgical excision of the osteophytes through an anterior cervical approach is reserved for persistent or severe cases [13,14]. More complex procedures, such as anterior cervical discectomy and fusion with osteophyte resection, are described mainly in patients with concurrent cervical myelopathy or spinal instability [8]. An additional treatment after surgical resection is referred to as the cyclic administration of etidronate disodium (1000 mg/day), which has been associated with the inhibition of ossification anterior longitudinal ligament [15].

Aim

The aim of the present study is (i) to describe three cases of progressive dysphagia secondary to cervical DISH, emphasizing their clinical presentation, diagnostic work-up, and management, and (ii) to review the recent literature (2015-2025) on this uncommon but clinically relevant complication. By doing so, we seek to underline the likelihood of underdiagnosis and to stress the importance of early recognition of dysphagia as a potential manifestation of cervical DISH.

## Case presentation

Clinical case 1

Presentation

An 82-year-old male presented to the emergency department with rapidly progressive dysphagia to both solids and liquids over the preceding month. He was unable to tolerate even soft foods and refused nasogastric tube placement. His medical history was significant for chronic renal failure, which had recently deteriorated due to malnutrition, and for excessive use of non-steroidal anti-inflammatory drugs (NSAIDs) for pharyngeal discomfort.

Key alert features: Elderly patient, short history of progressive dysphagia, normal upper airway endoscopy, and subsequent imaging showing large anterior cervical osteophytes compatible with DISH.

Examination

On physical examination, cervical range of motion was markedly restricted in all directions. Gait was normal, there were no signs of segmental or global instability, and the neurological examination of both upper and lower extremities was unremarkable. Hoffman and Tromner signs were negative, providing no clinical evidence of cervical myelopathy.

Investigations

**Figure 1:**
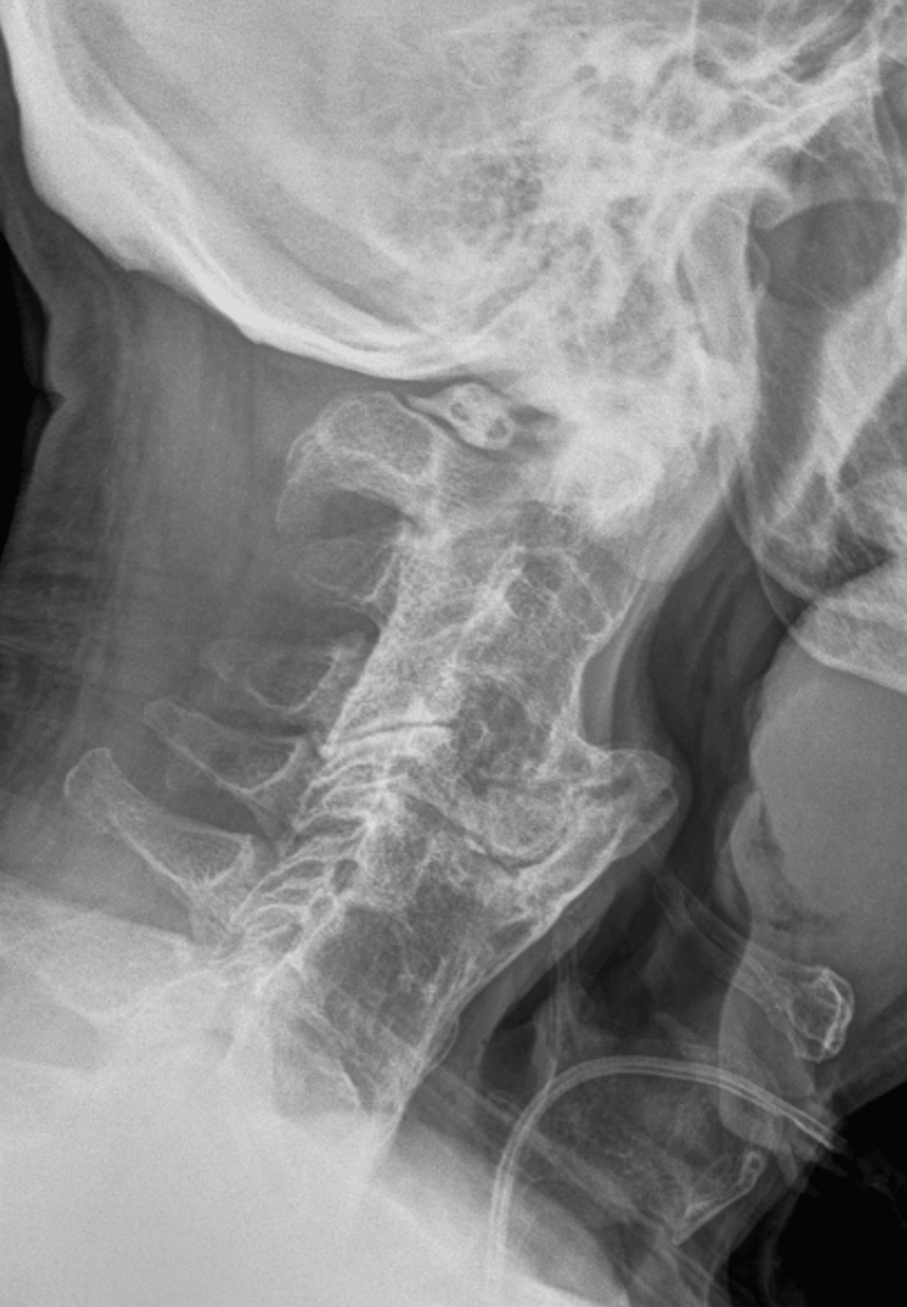
Clinical case 1. Lateral cervical radiograph showing prominent anterior osteophytes at C3–C5 levels, with effacement of the hypopharyngeal airway contour, compatible with dysphagia secondary to diffuse idiopathic skeletal hyperostosis (DISH).

**Figure 2:**
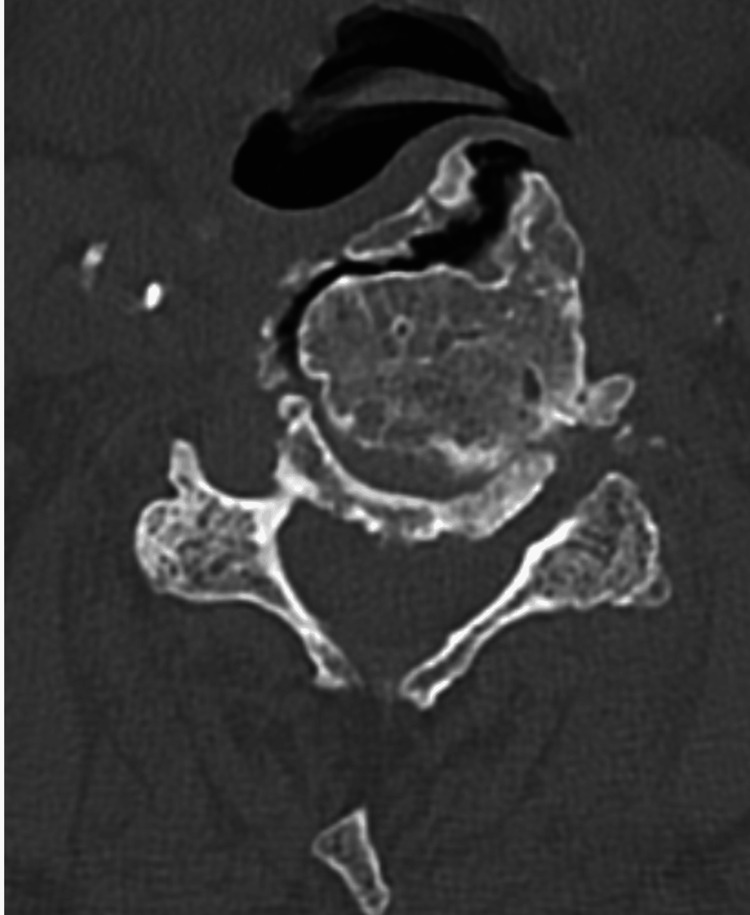
Clinical case 1. Sagittal CT anterior ossification along the cervical vertebral bodies consistent with diffuse idiopathic skeletal hyperostosis (DISH), with mass effect on the posterior wall of the pharynx.

**Figure 3:**
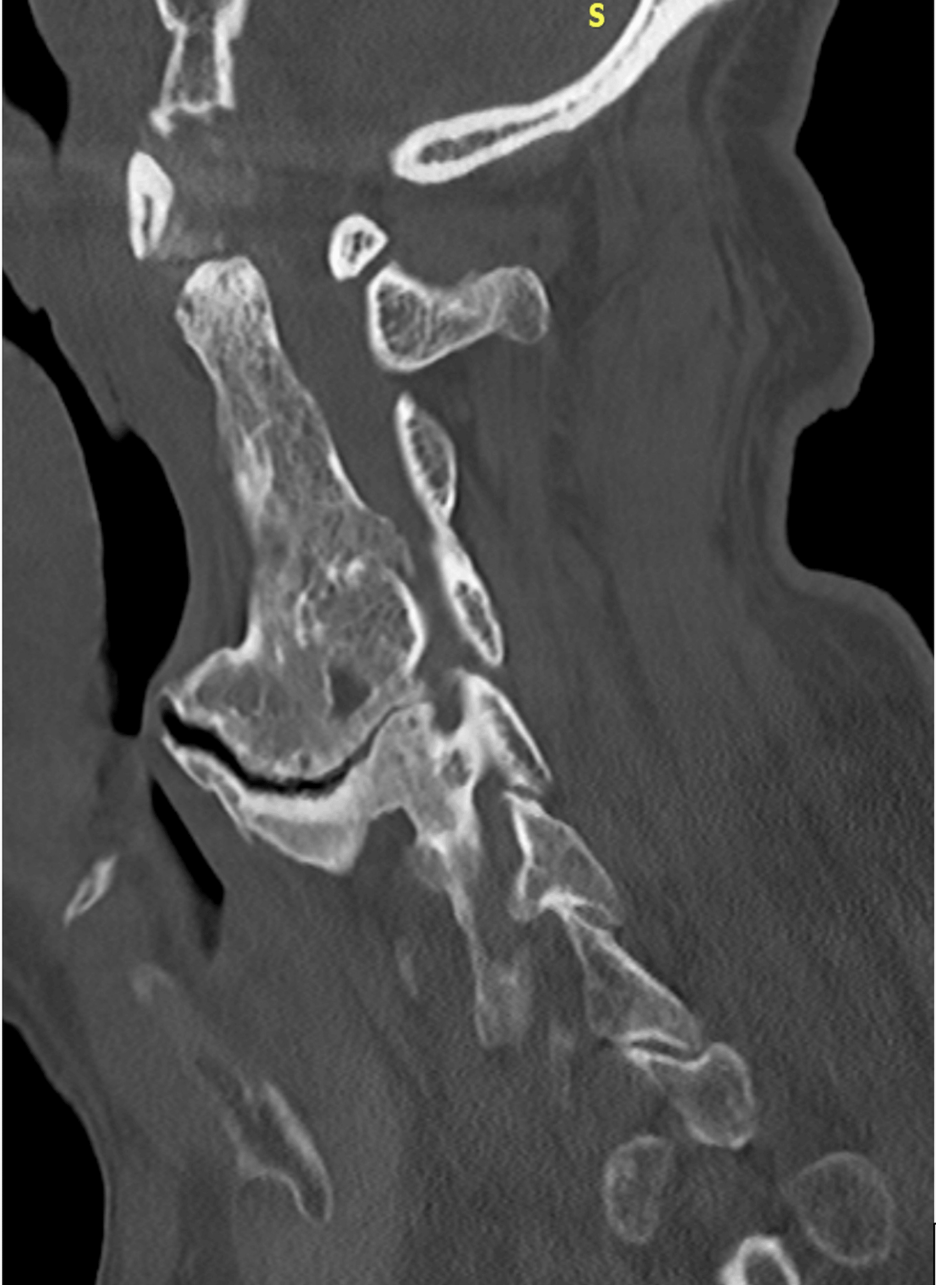
Clinical case 1. Axial CT of the cervical spine demonstrating bulky anterior osteophytes causing narrowing of the esophageal lumen.

Initial otolaryngology (ENT) assessment and upper airway endoscopy revealed no abscess, mass, or intraluminal obstruction. Because of persistent dysphagia despite normal endoscopic findings, cervical magnetic resonance imaging (MRI) was obtained and demonstrated calcification of the anterior longitudinal ligament with suspected esophageal compression. Plain anteroposterior and lateral cervical spine radiographs confirmed the diagnosis of DISH and showed a prominent anterior osteophyte at the C4-C5 level, causing posterior pharyngeal indentation, as illustrated in Figure 1. A barium swallow study demonstrated near-complete obstruction of the esophageal lumen, and cervical computed tomography (CT) was performed to further delineate the extent of the osteophytic formation. Figure 2 presents a sagittal CT scan, and Figure 3 represents an axial CT scan of the cervical spine.

Treatment

Given the severity of dysphagia and the radiological evidence of mechanical esophageal compression, the patient underwent surgical excision of the anterior cervical osteophytes through an anterolateral approach.

Outcome

**Figure 4:**
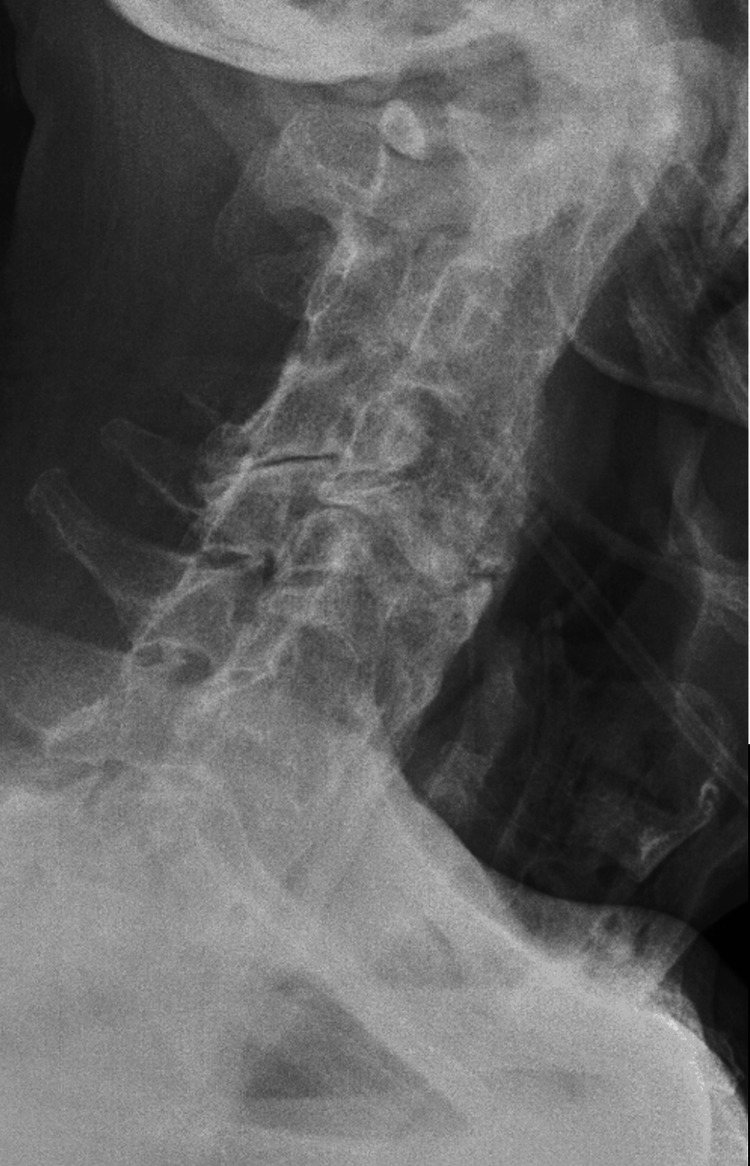
Clinical case 1. Postoperative oblique cervical radiograph after anterior cervical. No residual mass effect on anterior structures is seen.

Postoperative recovery was uneventful. At 40 days, repeat esophagogram and swallowing assessment confirmed the absence of residual material in the glottic region, and the patient resumed oral feeding without complications. At the 12-month follow-up, he reported complete resolution of dysphagia, was able to consume solid foods, and follow-up radiographs showed no evidence of osteophyte regrowth (Figure 4).

Clinical case 2

Presentation

A 78-year-old man presented to the emergency department with wheezing, which necessitated tracheostomy. He reported a one-year history of progressive dysphagia. His past medical history included Ménière’s disease and gastroesophageal reflux disease (GERD). A few days before admission, he had undergone laryngoscopic evaluation for dysphagia, which showed external compression of the larynx.

Key alert features: Elderly patient with long-standing progressive dysphagia, normal laryngoscopy but with external compression, and imaging later confirming large anterior cervical osteophytes due to DISH.

Examination

On physical examination, there were no neurological deficits in the upper or lower extremities. Hoffman and Tromner signs were negative, indicating no clinical evidence of cervical myelopathy.

Investigations

Following the laryngoscopic finding of external laryngeal compression, a cervical CT scan was obtained. CT demonstrated DISH of the cervical spine, with prominent anterior osteophyte formation extending from C3 to C6.

Treatment

Given the severity of dysphagia and the airway compromise that had led to tracheostomy, the patient underwent surgical excision of the anterior cervical osteophytes at the C3-C4 level via a left-sided anterolateral approach.

Outcome

At one month postoperatively, repeat laryngoscopy and esophagoscopy showed no residual compressive lesion in the glottic area, allowing for tracheostomy decannulation and closure. A postoperative cervical spine radiograph showed no recurrent osteophyte formation. At the 12-month follow-up, the patient remained asymptomatic, with no recurrence of dysphagia and no radiographic evidence of osteophyte regrowth.

Clinical case 3

Presentation

A 62-year-old man presented to the emergency department with a two-day history of deterioration in his general condition and altered level of consciousness. He had been bedridden for the previous two months because of generalized weakness and reported a one-year history of progressively worsening dysphagia. His medical history was notable for chronic kidney disease, diabetes mellitus, psychosis, chronic hyponatremia, former smoking, and reported alcohol abuse.

Key alert features: Adult patient with long-standing progressive dysphagia, normal initial laryngoscopic findings, and coexisting neurological signs later shown to be related to cervical DISH with posterior ligamentous calcification.

Examination

On admission, laboratory testing revealed severe hyponatremia, and the patient appeared malnourished, requiring nasogastric feeding. Neurological examination demonstrated findings suggestive of cervical myelopathy: weakness of the left upper limb, reduced muscle strength in the lower limbs, hyperactive deep tendon reflexes in all four extremities, and positive Hoffmann and Tromner signs.

Investigations

**Figure 5:**
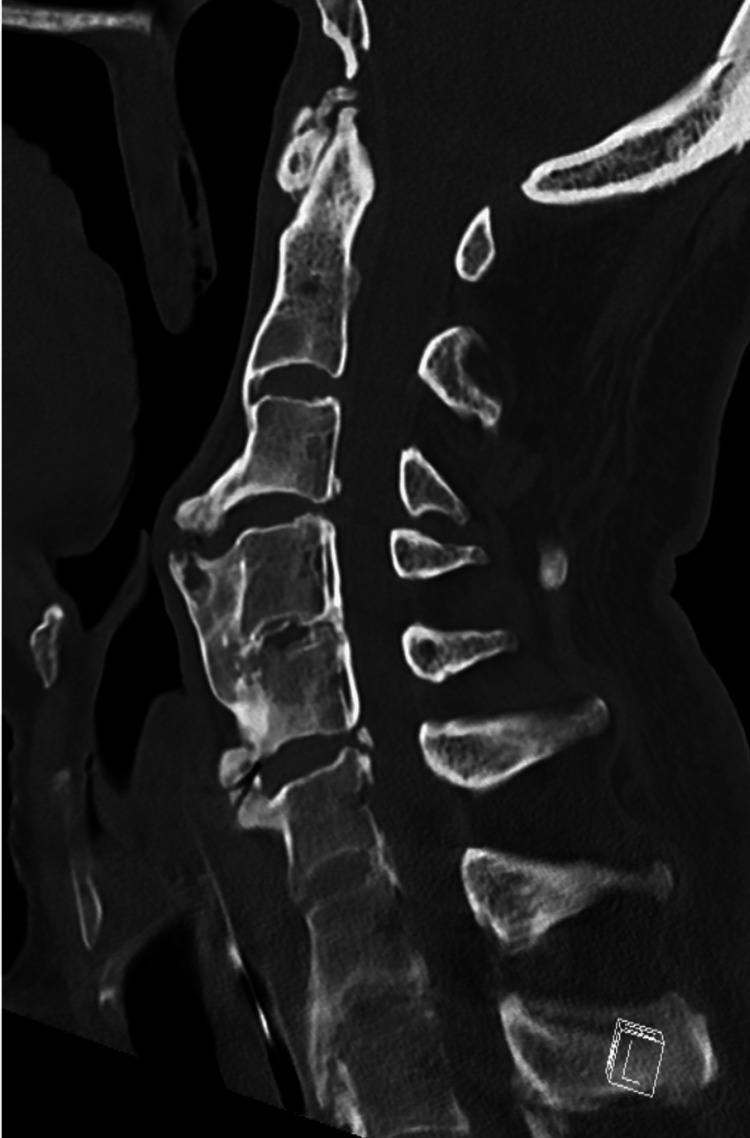
Clinical case 3. Preoperative sagittal CT showing the maximal craniocaudal extent of the anterior cervical osteophytes and their relationship to the airway and esophagus.

Because of his chronic kidney disease and electrolyte disturbance, he was admitted to the nephrology department, where gradual and closely monitored correction of hyponatremia was undertaken, and a nasogastric tube was placed for pureed nutrition. An otolaryngology consultation with direct laryngoscopy was performed as part of the dysphagia workup and showed no intraluminal compressive lesion. Given the neurological findings, neurology was consulted, and brain and cervical spine CT and MRI were obtained. Imaging revealed a large anterior osteophyte at the C3-C4 level and segmental calcification of the posterior longitudinal ligament from C3 to C5, producing mild central spinal canal stenosis with an anteroposterior dural sac diameter of approximately 9 mm, consistent with cervical involvement of DISH, as illustrated in Figure 5.

Treatment

In view of the severity of dysphagia and the documented mechanical anterior compression, the patient underwent surgical excision of the anterior cervical osteophytes at the C3-C4 level via a left-sided anterolateral approach. Because of his significant medical comorbidities and the need to minimize surgical risk, additional decompression for cervical myelopathy was deferred to a later stage after multidisciplinary discussion with the patient and his relatives.

Outcome

**Figure 6:**
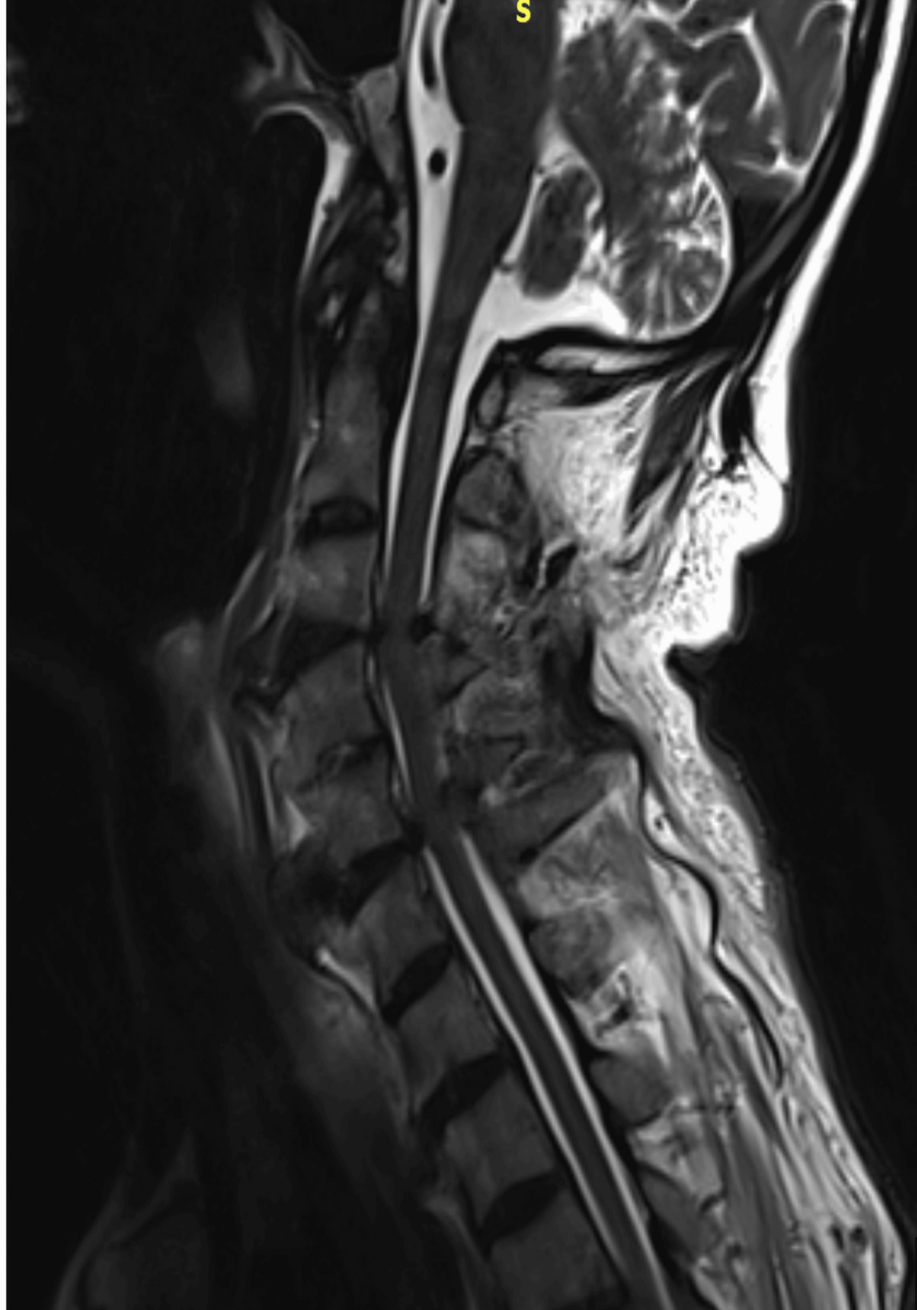
Clinical case 3. Postoperative lateral cervical radiograph after anterolateral osteophytectomy demonstrating removal of the obstructing anterior osteophytes.

At the three-month postoperative follow-up, cervical spine radiographs showed no evidence of recurrent osteophyte formation. Clinically, the patient reported near-complete resolution of dysphagia and was able to maintain oral intake. Figure 6 presents a postoperative lateral X-ray after performing anterolateral osteophytectomy, demonstrating removal of the obstructing anterior osteophytes.

## Discussion

Search strategy and literature review

This work consists of a retrospective description of three consecutive patients with dysphagia secondary to cervical DISH treated in our department, followed by a narrative, descriptive review of the literature. No formal systematic review, meta-analysis, or risk-of-bias assessment was performed.

**Figure 7:**
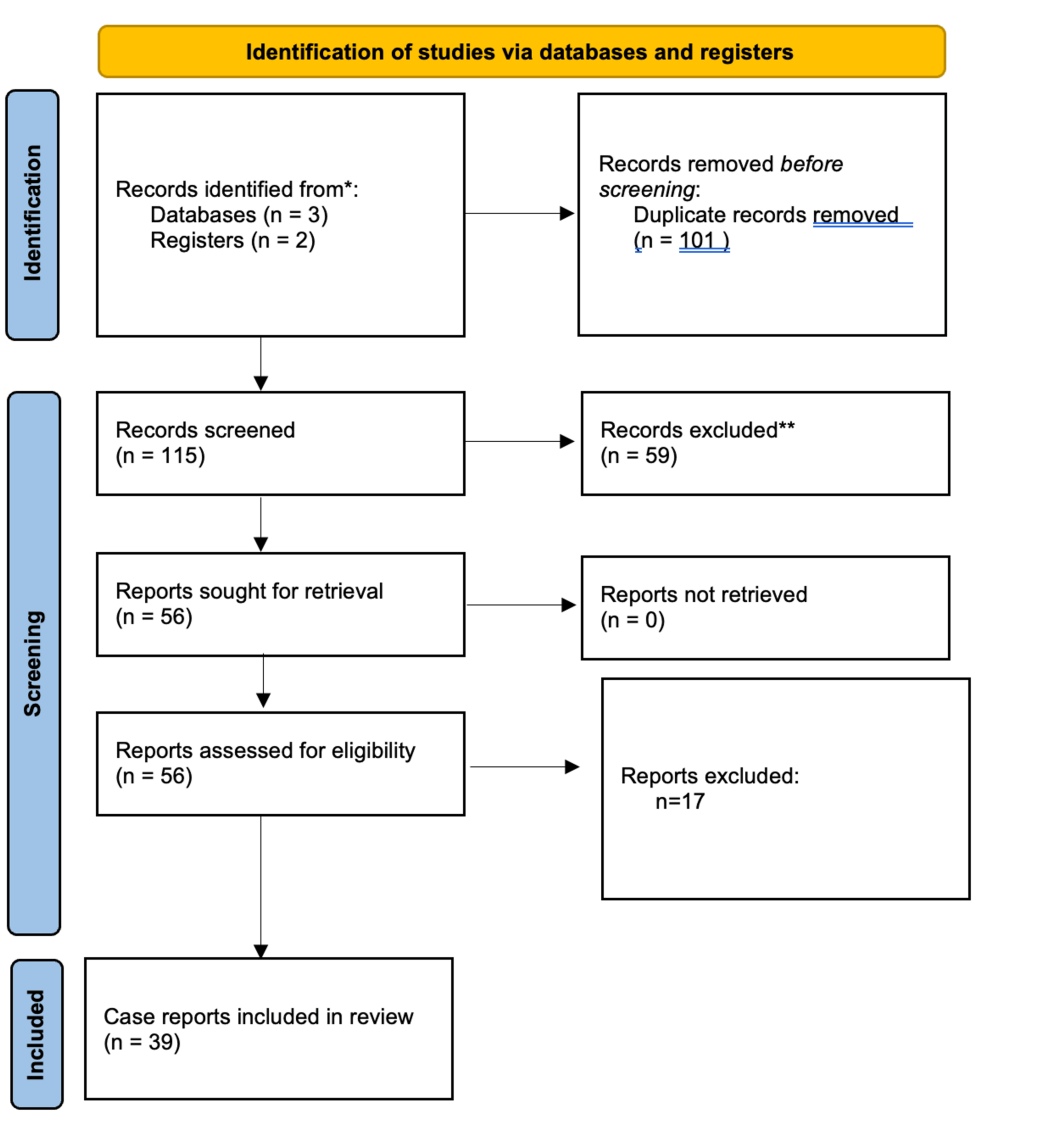
Flowchart.

Searches were performed in PubMed, Embase, and Cochrane Library using the keywords "Diffuse Idiopathic Skeletal Hyperostosis," "Forestier’s disease," and "dysphagia." Case reports published between 2015 and 2025 were included if they described cervical DISH presenting with dysphagia. Non-English papers and secondary causes of dysphagia were excluded. A flow diagram of included studies is presented in Figure 7.

Data analysis

Quantitative data from the published cases were summarized using basic descriptive statistics (counts, means/medians, and proportions). Percentages are reported as n/N (%) throughout the text and tables.

A total of 216 references were found, 101 duplicates were removed, and 59 articles were excluded based on title and abstract review. In addition, 56 articles were studied in detail, and 17 were excluded according to the exclusion criteria. Finally, 39 case reports were included in the study, concerning 44 patients who were included in the studies [7-12,15-47]. Table 1 summarizes clinical and management characteristics of published cases of cervical DISH with dysphagia.

**Table 1:** Case reports and patient characteristics. DISH: diffuse idiopathic skeletal hyperostosis; ACDF: anterior cervical discectomy and fusion; CT: computed tomography; ΜRI: magnetic resonance imaging; ENT: ear, nose, and throat; ROM: range of motion; GERD: gastroesophageal reflux disease.

Authors/year	Country	Age	Sex	Symptoms	Symptom duration (months)	Diagnosis modalities	Treatment	Comorbidities	Approach
Quaye et al. [16], 2015	UK	62	Male	Dysphagia, dysphonia, dyspnea	24	CT, dynamic contrast swallow study	Osteophytectomy without spinal fusion	Diabetes mellitus	n/a
Egerter et al. [17], 2015	USA	61	Male	Dysphagia, aspiration	36	CT	Osteophytectomy without spinal fusion	Appendicitis, gastroesophageal reflux disease, gout, hyperlipidemia	Anterior
		70	Male	Dysphagia, cervical pain, extremity weakness	240	Strobovideolaryngoscopy, dynamic fluoroscopy, X-ray, CT, MRI	Anterior C3–C7 osteophytectomy, diskectomy, decompressive laminectomy and C3–T3 instrumented fusion	Parkinson's disease, coronary artery disease, gastroesophageal reflux disease, depression	Anterior and posterior
Sugimura et al. [15], 2016	Japan	51	Male	Dysphagia recurrence 5 years post-op	n/a	Esophagography, CT, X-ray	Osteophytectomy without spinal fusion and etidronate disodium (800 mg/day for 6 months), revision osteophytectomy, and repeat etidronate disodium (1000 mg/day)	none	n/a
Srivastava et al. [18], 2016	India	60	Female	Dysphagia, weight loss	18	X-ray, CT, oesophagoscopy	Osteophytectomy without spinal fusion	n/a	Anterior
Allensworth et al. [19], 2016	USA	61	Male	Hoarseness, stridor, respiratory distress, dysphagia, weight loss	4	Laryngoscopy, X-ray, CT, MRI	Osteophytectomy without spinal fusion	Diabetes mellitus	Anterior (left anterolateral approach)
Ozdol et al. [20], 2015	Turkey	32	Male	Dysphagia, neurological radicular pain	24	X-ray, MRI, esophagography	C4-C6 microdiscectomies, cage-plate fusion	n/a	n/a
Stojanovic et al. [21], 2016	Serbia	69	Male	Dyspnea, dysphagia, hoarseness	2	CT, laryngoscopy	Conservative	n/a	n/a
Candelario et al. [22], 2017	USA	76	Male	Dysphagia, dysphonia, aspiration	n/a	MRI, CT	C4-C6 anterior approach, plate fixation, osteophytectomy, microdiscectomy	Alcohol abuse	Anterior (left side)
Kaur et al. [10], 2017	India	63	Male	Dysphagia, hoarseness, radicular pain	3	CT, MRI, X-ray	Osteophytectomy without spinal fusion	n/a	Anterior
Goico-Alburquerque et al.[23], 2017	USA	80	Male	Sore throat, odynophagia, cough, dyspnea, weight loss	6	Barium study, CT, laryngoscopy	None (decision for osteophytectomy was left to be discussed with the primary care physician after discharge)	Diabetes mellitus, smoking	n/a
Sinha et al.[9], 2017	Iran	49	Male	Dysphagia, weight loss	24	Barium, CT, X-ray, endoscopy	Conservative	Arterial hypertension, diabetes mellitus	n/a
Saffo et al. [24], 2017	USA	74	Male	Dysphagia, hypoxia	n/a	CT	Osteophytectomy without spinal fusion	n/a	Anterior
Hoey et al. [25], 2017	UK	77	Male	Dysphagia, dyspnea, apnea	3	Nasendoscopy, CT	Osteophytectomy, tracheostomy	n/a	Anterior
Karaarslan et al. [26], 2017	Turkey	77	Male	Dysphagia, weight loss, dyspnea	6	CT, MRI	Osteophytectomy without spinal fusion	n/a	Anterolateral
Butler et al. [27], 2019	USA	83	Male	Dysphagia	n/a	ENT consultation, X-ray, barium swallow	Conservative	GERD, arterial hypertension, chronic kidney disease	n/a
Sebaaly et al. [28], 2018	Canada	76	Male	Dysphagia, dyspnea, dysphonia	5	ENT consultation	Osteophytectomy without spinal fusion	n/a	Anterior
Psychogios et al. [29], 2018	Greece	82	Male	Dyspnea, dysphagia, stridor, weight loss	3	Laryngoscopy, CT, panendoscopy	Osteophytectomy without spinal fusion	Arterial hypertension, prostate hyperplasia, smoking, alcohol abuse	Anterior, (anterolateral extrapharyngeal)
Alsalmi et al. [30], 2018	Saudi Arabia	66	Male	Dysphagia, odynophagia, hoarseness	n/a	CT	Osteophytectomy (C2-C7) without spinal fusion	Diabetes mellitus, hypercholesterolemia, hemorrhagic rectocolitis, benign prostatic hypertrophy	Anterior (semi-vertical paramedian cervical)
Soejima Yu et al. [11], 2019	Japan	66	Male	Dysphonia, dysphagia, cervical myelopathy	2	CT, MRI, ENT consultation	Osteophytectomy with discectomy C3/C4 and fusion	Arterial hypertension, brain infarction, arrhythmia	Anterior
Dabrowski et al. [31], 2020	Poland	73	Male	Dysphagia	6	X-ray, CT, MRI, gastroscopy	Osteophytectomy without spinal fusion	Diabetes mellitus, arterial hypertension, paroxysmal atrial fibrillation	Anterion (Smith Robinson)
Lee et al. [7], 2020	Korea	65	Male	Dysphagia, odynophagia, weight loss, aspiration	5	ENT consultation, laryngoscopy, CT	Osteophytectomy without spinal fusion	Arterial hypertension, hyperlipidemia	Anterior
		64	Male	Dysphagia, hoarseness	3	ENT consultation, endoscopy, CT, X-ray	Osteophytectomy without spinal fusion	Paraparesis (3/5), hypoesthesia below T4 due to spinal cord injury, diabetes mellitus, hypercholesterolemia	Anterior
		82	Male	Dysphagia	n/a	ENT consultation, endoscopy, CT	Osteophytectomy without spinal fusion	Diabetes mellitus, arterial hypertension	Anterior
Legaye et al. [32], 2020	Belgium	72	Male	Dysphagia, back pain	n/a	ENT consultation, endoscopy, X-ray, CT	Osteophytectomy without spinal fusion	Hyperlipemia, hypoparathyroidism	Anterolateral approach
Gao et al. [33], 2020	China	59	Male	Dysphagia, weakness in his left limbs	6	Esophagography, MRI, CT	Osteophytectomy with fusion	Coronary atherosclerotic heart disease	Anterior
Kumar et al. [34], 2020	India	56	Male	Dysphagia, dysphonia	12	CT, barrow swallow, MRI	Osteophytectomy without spinal fusion	n/a	Anterior
Jiao et al. [35], 2020	China	54	Male	Pharyngeal discomfort	½ month	Barium esophagogram, CT	Conservative	Diabetes mellitus	
Zarei et al. [36], 2020	Iran	68	Male	Dysphagia, stridor, dysphonia, decreased ROM of the cervical spine	12	Video-fluoroscopy, X-ray, CT, MRI	Osteophytectomy without spinal fusion	n/a	Anterolateral approach
Liawrungrueang et al. [8], 2021	Thailand	48	Male	Dysphagia, upper extremity weakness, cervical myelopathy	6	MRI, ENT consultation	Anterior Cervical Discectomy and Fusion (ACDF) with plate fixation and osteophytectomy	Arterial hypertension	Anterior (Smith-Robinson)
Mahajan et al. [12], 2021	India	69	Male	Dysphagia, upper extremity weakness, cervical myelopathy	4	CT, MRI	Posterior laminectomy, Decompression, and fusion	n/a	Posterior
Choi et al. [37], 2022	Korea	64	Male	Dysphagia in solid food absorption when swallowing liquid	2	Laryngoscopic examination, X-ray, CT, visual fluoroscopic swallowing study (VFSS)	Osteophytectomy without spinal fusion	Atrial fibrillation	Anterolateral approach
	Korea	65	Male	Dysphagia in liquid and solid, odynophagia, weight loss	3	Laryngoscopic examination, X-ray, CT, visual fluoroscopic swallowing study (VFSS)	Osteophytectomy without spinal fusion	Stomach cancer, bipolar disorder	Anterior (modified Smith–Robinson)
Kosmidou et al. [38], 2022	Greece	69	Male	Inspiratory stridor, dyspnea, difficulty in swallowing liquids and solids, hoarseness	12	CT, laryngoscopy	Osteophytectomy without spinal fusion	Prostatectomy, total hip replacement	Anterior
Reigota et al. [39], 2023	Portugal	70	Male	Dysphagia, dysphonia, weight loss	3	ENT consultation, CT, barium swallow	None (reference of the patient for orthopedic consultation for evaluation for possible surgery)	Parkinson ’s disease	n/a
Soares et al. [40], 2023	Portugal	84	Male	Dysphagia, foreign body sensation, weight loss	12	X-ray, CT, brain MRI, dynamic swallowing study with barium contrast	Osteophytectomy without spinal fusion	Arterial hypertension, degenerative aortic valvopathy, suspicion of early dementia	Anterior (Smith-Robinson)
Mesolella et al. [41], 2023	Italy	63	Male	Dysphagia, dysphonia, aspiration, cervical pain, swelling of the preauricular region	12	CT, MRI, gastroendoscopy, laryngoscopy	Conservative	Diabetes mellitus, arterial hypertension, hypercholesterolemia, smoking	n/a
Al-Jafari et al. [42], 2023	Jordan	50	Male	Dysphagia, cervical pain	72	CT	Osteophytectomy without spinal fusion	Diabetes mellitus, arterial hypertension, cerebrovascular accident, hyperlipidemia	Anterior
Avrumova et al. [43], 2024	USA	68	Male	Dysphagia to solids and liquids	Acute	CT, X-ray, dynamic fluoroscopy	Anterior osteotomy with computer navigation	Arterial hypertension, dyslipidemia	Anterior
Lyrtzis et al. [44], 2024	Greece	85	Male	Dysphagia	24	Endoscopy, CT, ENT consultation, fiberoptic endoscopic evaluation of swallowing (FEES), X-ray	Conservative	Arterial hypertension, diabetes mellitus	n/a
Balta et al. [45], 2024	Turkey	66	Male	Dysphagia, weight loss, cervical myelopathy	12	CT	Anterior decompression (C3-C5) with posterior decompression and laminectomy	None	Anterior and posterior
Pruijn et al. [46], 2024	Netherlands	78	Male	Dysphagia, hoarseness, weight loss, dyspnea	6	CT, laryngoscopy	Osteophytectomy without spinal fusion	Appendicitis, gastroesophageal reflux disease, gout, hyperlipidemia	Anterior
	Netherlands	82	Male	Dysphagia, dyspnea	n/a	CT, laryngoscopy	Osteophytectomy (C3–C7) with diskectomy and decompressive laminectomy and instrumented arthrodesis (C3–T3)	Parkinson’s disease, coronary artery disease, gastroesophageal reflux disease, depression	Anterior and posterior
Magno et al. [47], 2025	Portugal	70	Male	Dyspnea, cough, hoarseness, odynophagia	6	MRI, ENT, gastroenterologist consultation	n/a (the patient was stabilized and transferred to the tertiary care center)	Arterial hypertension, hyperlipidemia	Anterior approach

Patient demographics

The mean age of the patients was 67.3 years. The youngest reported patient was 32 years old [26], whereas the oldest was 85 years old [36]. There was a clear male predominance, with 42/44 patients (95.5%) being male. Most of the reported cases originated from Asian countries. Symptom duration, when reported, had a mean of 18.3 months, indicating a relatively long pre-diagnostic interval.

Most frequent symptoms and medical history of patients

**Figure 8:**
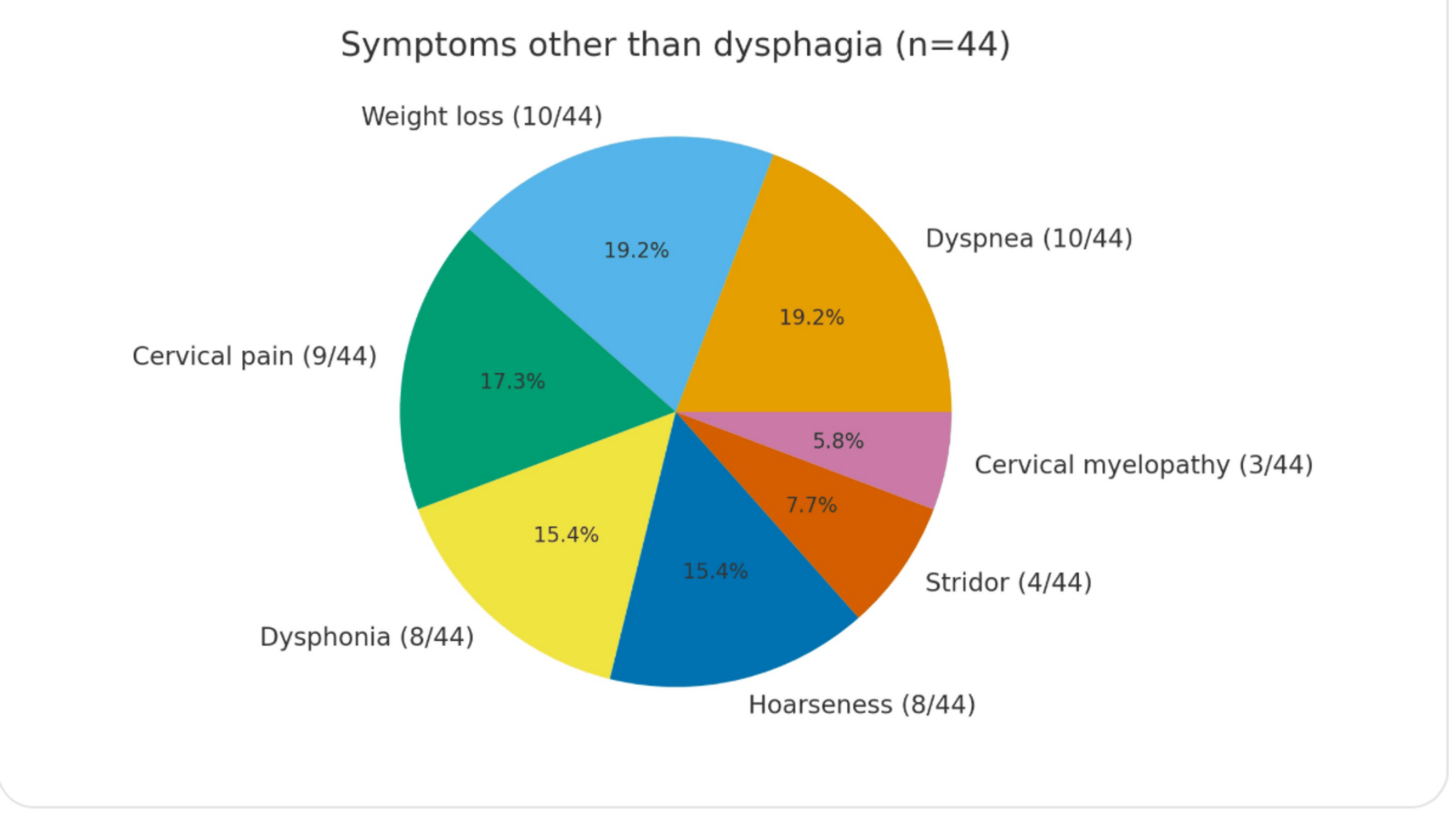
A pie chart representing the most frequent symptoms, apart from dysphagia.

Among the 44 patients, dysphagia was reported in all patients. Dyspnea was the second most frequent presenting complaint, though less prevalent. The third presented symptom was weight loss due to difficulty in consuming food in an easy way. The rest of the presented symptoms include cervical pain and tenderness, hoarseness, and dysphonia, which are equally presented. Finally, cervical myelopathy is presented as an independent symptom in some cases and without a pathophysiological relation to the formation of anterior cervical osteophytes. Table 2 summarizes the frequency and percentage of the main presenting symptoms among the patients included in this series, and Figure 8 represents the most frequent symptoms, apart from dysphagia.

**Table 2:** Distribution of presenting symptoms in reported patients.

Symptom	No. of patients	Frequency (%)
Dysphagia	44	100% (44/44)
Dyspnea	10	22.7% (10/44)
Weight loss	10	22.7% (10/44)
Dysphonia	8	18.2% (8/44)
Cervical pain	9	20.4% (9/44)
Hoarseness	8	18.2% (8/44)
Cervical myelopathy	3	6.8% (3/44)
Stridor	4	9.1% (4/44)

Surgical approach

**Figure 9:**
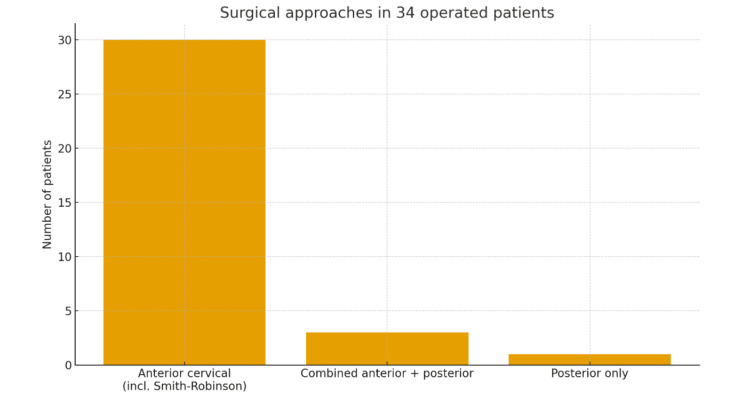
Bars showing the most preferred surgical approaches.

Among the 44 patients, 34/44 (77.3%) underwent surgical treatment. Among those who were operated on, the anterior cervical approach was clearly the preferred technique, being used in 30/34 (88.2%) of surgically treated cases. In this review, the term “anterior cervical approach” denotes any approach to the cervical spine through the anterior aspect of the neck; when specifically reported, the Smith-Robinson approach is mentioned, which is performed through the plane between the sternocleidomastoid and the strap muscles, with lateral retraction of the carotid sheath and medial retraction of the trachea and esophagus. A smaller subset was managed with a combined anterior-posterior approach (3/34, 8.8%), whereas a posterior-only approach was selected in 1/34 (2.9%) due to concomitant cervical myelopathy. Figure 9 illustrates the distribution of the three surgical strategies, confirming the predominance of the anterior approach.

Comorbidities in reported cases

The most common comorbidities observed among patients in the reported cases included arterial hypertension, diabetes mellitus, hyperlipidemia, and gastroesophageal reflux disease, with additional occurrences of atrial fibrillation, coronary artery disease, Parkinson’s disease, and various psychiatric, metabolic, and cardiovascular conditions.

Diagnosis

Regarding diagnostic evaluation, 17/44 (38.6%) patients underwent plain radiography of the cervical spine, whereas 39/44 (88.6%) had a cervical CT scan. Plain radiographs provide a simple, rapid, and low-cost method to detect anterior cervical osteophyte formation in symptomatic patients; however, because clinical suspicion for DISH-related dysphagia is often low, many patients undergo additional specialized investigations. These included laryngoscopy, videofluoroscopic swallowing study (VFSS), barium swallow, oesophagoscopy, and nasoendoscopy, performed according to presenting symptoms and the referring specialty.

Follow-up and complications

Follow-up was variably reported across the 44 published cases, preventing a uniform pooled analysis. In those patients who underwent anterior cervical osteophytectomy, the postoperative course was generally favorable, with resolution or marked improvement of dysphagia in most reports. A single case of late recurrence was described five years after the index surgery, requiring repeat osteophytectomy and adjuvant etidronate therapy, indicating that regrowth of osteophytes is possible in the long term. A small number of procedure- or disease-related complications were reported, including airway compromise requiring tracheostomy and one fatal outcome secondary to postoperative ulcer infection. Because outcome reporting was inconsistent among studies, these data are presented descriptively rather than quantitatively, and should be interpreted with caution.

Discussion

DISH, also referred to as Forestier’s disease, is a non-inflammatory systemic condition characterized by calcification and ossification of spinal ligaments and enthuses. The most commonly affected ligament is the anterior longitudinal ligament [48]. It predominantly affects men over the age of 50 and is frequently asymptomatic. However, it can lead to significant complications such as dysphagia, stridor, aspiration pneumonia, and, in rare instances, upper airway obstruction [47]. Dysphagia associated with DISH is uncommon and often underrecognized. Esophageal compression due to anterior cervical osteophytes, also termed cervical osteophyte-induced dysphagia (COD), is estimated to account for approximately 1.7% of all dysphagia cases [6]. When present, symptoms may range from mild difficulty in swallowing solids to complete intolerance of liquids, as demonstrated in our first case.

The cervical spine levels most frequently implicated are C3-C5. These levels correspond anatomically to the narrowest segment of the hypopharynx and upper esophagus. This anatomical correlation aligns with our findings, where C3-C4 involvement was observed in the first patient and C4-C5 in the second.

An important factor contributing to the development of dysphagia is the size of the anterior cervical osteophyte. In a retrospective study by Strasser et al. [6], both increased patient age and larger osteophyte size were independently associated with a high risk of aspiration. Aspiration was observed in 75% of patients with osteophytes larger than 10 mm, compared to 34% of those with osteophytes measuring 10 mm or less. Similarly, another study by Seidler et al. [49] reported that osteophytes smaller than 5 mm in anteroposterior dimension were not associated with swallowing impairment, whereas osteophytes exceeding 10 mm were consistently linked to symptomatic dysphagia. In the present case series, the mean anteroposterior size of the osteophytes measured up to 31 mm, supporting the correlation between increased osteophyte size and symptom severity.

In addition to the clinical presentation, imaging plays a pivotal role in the diagnosis of DISH. Although initial assessments may include esophagoscopy, laryngoscopy, or a barium swallow study, definitive diagnosis requires imaging of the cervical spine. Typically, plain radiographs, CT, or MRI of the cervical spine can help to identify anterior cervical osteophytes formation. In both of our cases, CT imaging confirmed the presence of prominent anterior cervical osteophytes consistent with DISH. Nevertheless, plain cervical spine radiography remains the most accessible, rapid, and cost-effective initial imaging modality for detecting anterior osteophytes.

Although many patients with DISH remain asymptomatic, the presence of severe dysphagia or associated complications such as malnutrition, aspiration, or airway obstruction necessitates a comprehensive assessment. Conservative treatment, which includes dietary modification, anti-inflammatory medication, and swallowing therapy, may be the appropriate treatment for patients with mild symptoms. On the other hand, surgical treatment is indicated in cases where the conservative measures prove ineffective or when symptoms progress to a severe or debilitating level.

Surgical treatment includes the surgical resection of anterior cervical osteophytes. In the published cases, the anterolateral/anterior cervical approach was the most commonly reported technique for osteophyte excision. In most reported cases, this approach was chosen as it allows direct exposure of the osteophytes with relatively limited soft-tissue disruption [22]. In our three cases, this approach was performed. Nonetheless, meticulous surgical technique is essential, given the proximity of vital neurovascular and aerodigestive structures, which pose a risk for intraoperative complications. The primary significance of surgical excision lies in its potential to provide rapid symptomatic relief. According to the available case reports and small series, swallowing function improved markedly after surgery. In patients without neurological deficits, such as those presented here, excision of the osteophytes alone is generally sufficient. However, in the presence of cervical myelopathy or spinal cord compression, additional surgical procedures, such as anterior cervical discectomy and fusion (ACDF) or posterior decompression, may be necessary [8]. These management considerations should be interpreted with caution, as they are based on a small case series and descriptive reports.

Our three cases also highlight specific clinical challenges encountered in the management of DISH. The first involved a patient with advanced chronic kidney disease and severe malnutrition, which complicated both the diagnostic evaluation and perioperative care, whereas the second case illustrated a rare but serious complication, such as airway compromise, which required tracheostomy. This emphasizes the potential severity of DISH-related COD. Finally, our third case involved a patient with chronic kidney disease and severe hyponatremia, which required careful imaging evaluation and tailored surgical planning to balance symptom relief vs overall health status.

Overall, DISH-related COD appears to be underreported in Western populations. This may in part reflect publication bias and limited reporting from European centers. Dutta et al. [50] observed a predominance of reported cases from Asia, with significantly fewer documented in European countries. This report represents one of the few confirmed cases in Greece, emphasizing the need for heightened clinical awareness. This awareness stands even higher, particularly in regions where the condition may be underdiagnosed or misattributed to more common causes of dysphagia.

Surgical outcomes for DISH-related dysphagia are generally favorable, with most patients experiencing significant and sustained symptom relief. Although postoperative recurrence is uncommon, long-term follow-up is essential to monitor for potential osteophyte regrowth. In our cases, all three patients remained asymptomatic at the one-year follow-up, with no radiological evidence of recurrence.

## Conclusions

Cervical DISH is an underrecognized but treatable cause of secondary dysphagia in older adults. In our series, targeted imaging identified large anterior cervical osteophytes (predominantly C3-C5), and anterolateral osteophytectomy yielded rapid and durable symptom resolution. Our literature review (39 reports; 44 patients) reinforces that dysphagia is the dominant presentation, that diagnostic delays are common due to low clinical suspicion, and that conservative measures suffice only for mild disease. When malnutrition, airway compromise, or failure of conservative therapy is present, early surgical referral should be prioritized; in the absence of myelopathy, osteophytectomy alone is usually adequate, whereas concomitant cord compression warrants decompression and/or fusion. Postoperative recurrence appears uncommon, but longitudinal follow-up is advisable to monitor for regrowth and to optimize swallowing outcomes. Heightened awareness among primary care, ENT, gastroenterology, and spine services can shorten time to diagnosis and improve patient nutrition, safety, and quality of life.
